# The Influence of Customized Mouthguards on the Muscular Activity of the Masticatory Muscles at Maximum Bite and Motor Performance During Static and Dynamic Exercises

**DOI:** 10.1186/s40798-021-00354-2

**Published:** 2021-09-03

**Authors:** Johannes Lässing, Christoph Pökel, Lennart Lingener, Roberto Falz, Stefan Kwast, Antina Schulze, Martin Busse

**Affiliations:** 1 Institute of Sports Medicine and Prevention, University of Leipzig, Marschnerstr. 29, 04109 Leipzig, Germany; 2 SC DHfK Leipzig Handball, Leipzig, Germany

**Keywords:** Concurrent activation potentiation, Remote voluntary contraction, Performance enhancing effects, Improved activation symmetry

## Abstract

**Background:**

Some studies have suggested that a mouthguard is a performance-enhancing device due to a remote voluntary contraction. The extent to which a mouthguard can induce this phenomenon, e.g., by potentially increasing biting, has not been clarified. This study’s aim was to investigate the muscular activity of the maxillary and peripheral musculature and motor performance during a rest and exercise test.

**Methods:**

Our study comprised 12 active, male, professional young handball players (age 18.83 ± 0.39 years). Their performance, electromyographic (EMG) muscle activity (*Σ*), and lateral deviation (*Δ*) of the masticatory and peripheral musculature were measured during rest in a maximum bite force measurement, one-legged stand, a kettlebell swing exercise and a jump test while wearing a customized mouthguard (CMG) or not wearing one (Co).

**Results:**

Maximum bite force measurements did not differ significantly in their mean values of muscle activity (*Σ*) for the masseter and temporalis muscles (Co 647.6 ± 212.8 µV vs. CMG 724.3 ± 257.1 µV *p* = 0.08) (Co 457.2 ± 135.5 µV vs. CMG 426.6 ± 169.3 µV *p* = 0.38) with versus without CMG. We found no differences in the mean activation values during a one-legged stand, the kettlebell swing, and jump test (*Σ*) in any of the muscles tested. Lateral deviations (*Δ*) wearing a CMG were significantly less in the erector spinae during the kettlebell swing (Co 5.33 ± 3.4 µV vs. CMG 2.53 ± 1.8 µV *p* = 0.01) and countermovement jump (Co 37.90 ± 30.6 µV vs. CMG 17.83 ± 22.3 µV *p* = 0.03) compared to the performance without a CMG. Jump height, rotation moment, and balance were unchanged with versus without CMG.

**Conclusion:**

Our results at rest and during specific motor stress show no differences with or without a CMG. The improved peripheral muscular balance while wearing a CMG indicates improved muscular stabilization.

## Key Points

- The protective effects of mouthguards has been adequately documented. Some studies suggest that the use of customized mouthguards can have partial performance-enhancing effects.
- No poorer or better motor performance was observed when using a customized mouthguard.
- No increased sEMG-induced activation of the masseter or peripheral muscles was observed with a customized mouthguard.
- EMG-induced muscle activation resulted in more balanced control of the back muscles during dynamic exercises when using a customized mouthguard.

## Introduction

Jørgensen [27] reported a prevalence of 8.3 maxillofacial injuries per 1000 h in handball sports. In a study of children playing handball, Galic et al. [20] found dental injuries in 21.8% of all children and estimated the risk of dental injury to resemble that associated with the martial art of karate. Some studies have noted that just 5.7 to 14.5% of interviewed handball players wear mouthguards consistently [20, 29, 36]. There is evidence that CMGs do not detract from or influence an athlete’s performance, cardiopulmonary parameters, or oxygen uptake (VO_2_) [7, 28, 31, 32, 39]. A few studies have shown that teeth clenching plays an important role in rapid postural stabilization [19, 25, 33], gait stabilization [18], and balance control [4]. Some investigations [26, 40] detected positive correlations between a modified occlusal vertical dimension and the head, as well as a cervical posture in patients affected by craniomandibular disorders, while others failed to demonstrate a relationship between an occlusal change and posture [35].

However, numerous studies have demonstrated performance-enhancing effects when mouthguards are worn [2, 6, 13, 21, 34, 37–39]. Some authors have suspected that performance improvements can be achieved when wearing an mouthguard compared to without [3, 6, 9, 13, 16, 34, 37, 38]. Referring to the research of Ebben et al. [15, 16], the majority of authors promote the mouthguard-induced concept of concurrent activation potentiation (CAP) [2, 3, 6, 9, 13, 34, 38]. The CAP mechanism has a spectrum of theoretical backgrounds associated with potentiation-phenomenon mechanisms [14] and with intercortical communication behavior [11]. This mechanism of force potentiation is partially attributable to a pre-activated motor cortex, that is, increased bite force with an mouthguard, resulting in remote voluntary contraction (RVC) of the temporomandibular joint and consequent activation of other motor regions located in the cortex [14, 16]. There is some evidence of improved power output during ergometry [3, 34] and increased power output during a vertical jump (CMVJ) [1, 2, 13] while wearing an mouthguard. Analogous to these demonstrably increased effects, mouthguards wear also appears to promote motor pre-activation via the manipulated jaw muscles, which in turn may have a forced effect on the associated larger muscle groups [1, 2, 6, 14–16, 34, 38, 39].

Although most investigations have assumed a forced bite with mouthguards [1, 6, 34], we have not found adequate evidence for this assumption. The present study investigates the theory of increased activation of the jaw muscles due to wearing mouthguards and there relevance for peripheral activation of different muscle groups in dynamic and static loading tests with and without customized mouthguard (Co). Based on the known effects of wearing a mouthguard, improved EMG-induced muscle activity of the masseter muscles (*Σ*), peripheral muscle activity (*Σ*), and improved performance in sport-specific exercises should be expected for wearing a mouthguard.

## Materials and Methods

### Ethics Approval and Study Group

This study was approved by the Ethics Committee of the Medical Faculty of Leipzig University (445–15–21122015) and was conducted in accordance with the latest revision of the Declaration of Helsinki. Participants were excluded from the tests if they had any orthopedic, metabolic, cardiorespiratory diseases or temporomandibular disorders.

Written informed consent was obtained from all participants. The study comprised 12 young, active, male professional handball players. All participants are young handball league athletes who play for the A-youth of the first-division SC DHFK Leipzig team, and they train 10.5 h per week. Participants engaged in no physical exercise 24 h before the examinations and were required to consume 10 g of carbohydrates per kg body weight the day before to ensure their glycogen levels remained stable. The participants were instructed not to take stimulants such as energy drinks or caffeine.

### Mouthguard Production

The bite registration was made with a warm wax template of 4 mm thickness placed on the lower jaw. The lower jaw was guided into a repeated quick and easy relaxed slight bite impression of the upper teeth.

The customized mouthguard (CMG) was vacuum-formed over a stone model that had been made from a dental impression (alginate). A thermoformed plastic foil of 3 mm thickness was used as base material (Erkoflex, Erkodent Erich Kopp GmbH, Germany), and the thermoforming process was made with the Erkoform-3d motion device (Erkodent Erich Kopp GmbH, Germany).

The Occluform-3 device was used to imprint the opposing bite according to the bite registration.

## Study Design

In a prospective, randomized, crossover design, we investigated the effects on craniofacial muscle activation during maximum-bite measurements under resting conditions, as well as muscle activation under dynamic and static requirements, while a customized mouthguard (CMG) was worn compared to the procedure with no mouthguard (Co). Muscular stimulation, the activation distribution, and motor performance were assessed.

Our study was divided into two aspects (a rest examination and stress examination). The rest examination (RE) *took place on two different days*. At their first examination appointment (RE1- Serves only for measurement preparation), subjects were instructed about the study protocol and informed consent was obtained. A dental impression and bite registration was taken from the subjects to prepare the CMG.

The second examination appointment (RE2) consisted of a maximum bite measurement via the SINFOMED K7 system (SinfoMed, Frechen, Germany). In RE2, we measured the neuromuscular activities of the masseter and temporalis muscles (sEMG) during a block randomized maximum bite measurement with and without a mouthguard. No familiarization time for the mouthguards was provided for the examinations.

**Fig. 1:**
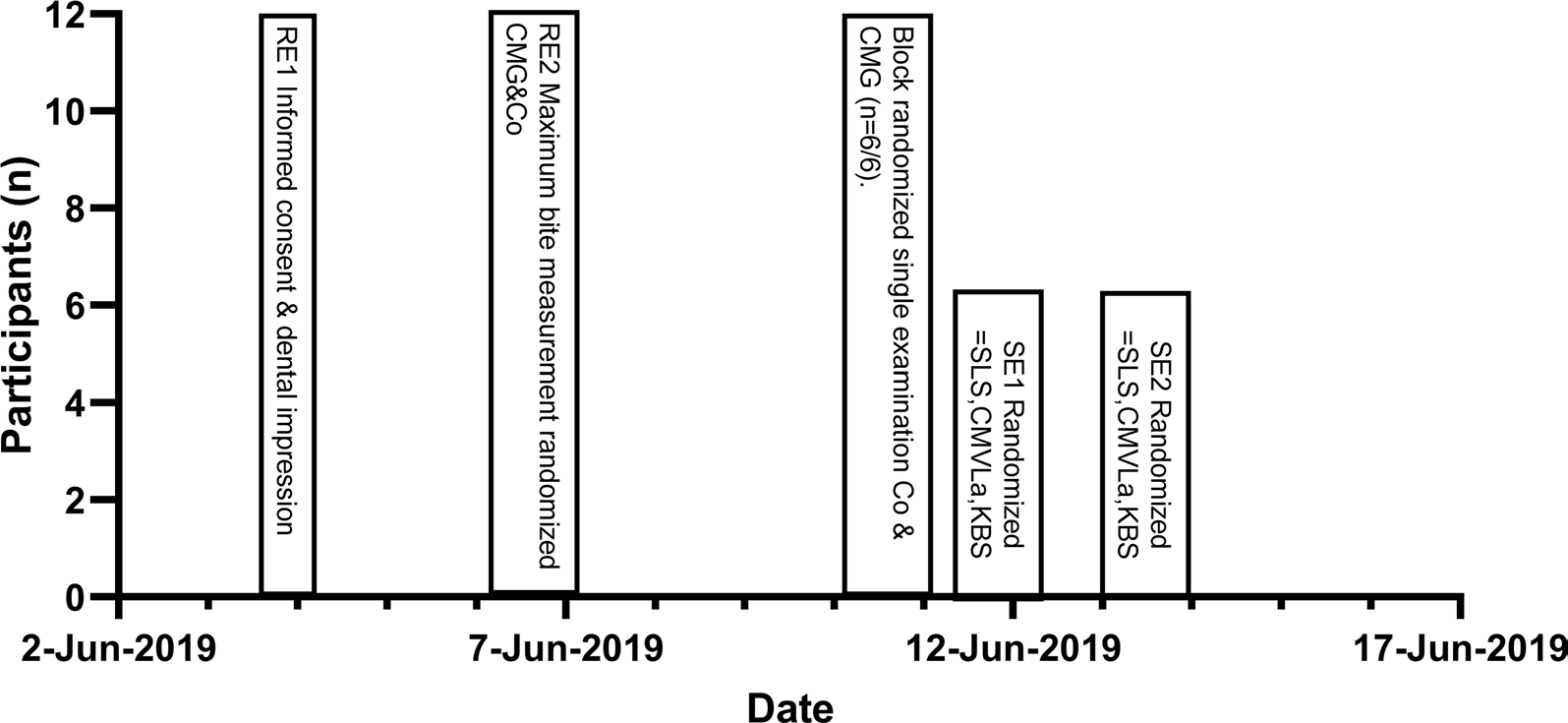
Examination procedure for RE1 = rest examination 1, RE2 = rest examination 2, SE1 = exercise test 1, SE2 = exercise test 2 (Co = without mouthguard, CMG = with mouthguard), CMVJa = countermovement jump with arms, KBS = kettlebell swing, SLS = single-leg stand

During the following exercise tests (SE), the single-leg stand (SLS), countermovement jump with arms (CMVJa), and kettlebell swing (KBS) were quasi-randomized for the block randomized conditions (CMG and without CMG). The test participants are professional athletes, and all the performed exercises are part of their training. These two test days (SE1/SE2) were exactly 24 h apart for each subject. Figure 1 shows the study design of the examinations.

### Maximum Bite Measurement

The myofunctional examination was *randomly* done with the SinfoMed K7 system (SinfoMed, Frechen, Germany). It measures neuromuscular activity using bipolar surface electrodes. For all tests, subjects were placed in a chair and required to assume a natural, upright, and relaxed position without head or neck support. The sEMG activities of the temporalis and masseter muscles were recorded bilaterally. The sEMG activity was recorded under the condition of maximum voluntary clenching (MVC) in intercuspal position. The subjects had to bite explosive and as hard as they could for two seconds. A 30-s break between the 2-s maximum pressing phases was maintained between clenches.

### Exercise Measurement

**Fig. 2:**
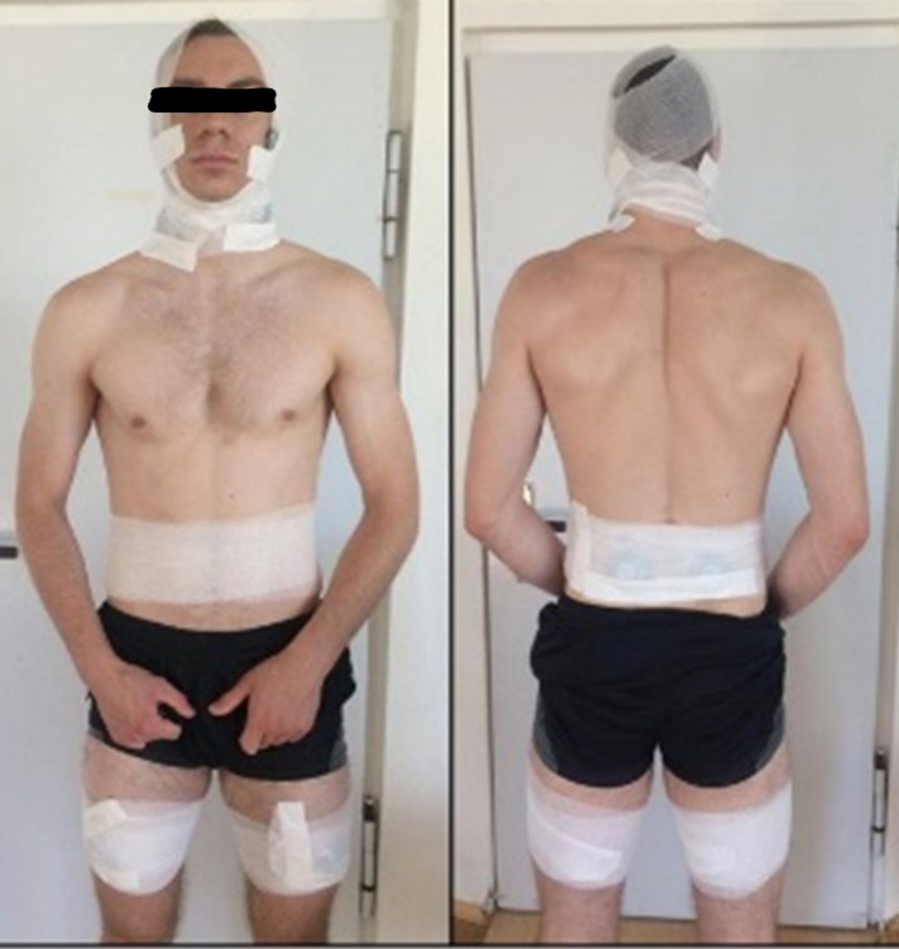
EMG preparation of the subjects

At the start of each measurement appointment (SE1/SE2), the athletes were instructed in a standardized process, and electrode marks were placed at anatomically defined fixed points [23]. The EMG electrodes were affixed on the person and fixed with cohesive conforming bandages (Peha-haft, PAUL HARTMANN AG, Germany). The EMG signals were recorded from the masseter, sternocleidomastoid, erector spinae lumbar (L4), and rectus femoris muscle groups. Electrode placement is based on recommendations for surface EMG to assess a non-invasive assessment of muscles tone (SENIAM) [23]. Study participants were instructed not to remove the markings that had been made with a skin marker. Figure 2 shows our subjects’ EMG preparation. They all signed a consent form that permits us to publish the photographic material in a journal.

### One-Legged Balance Test

**Fig. 3:**
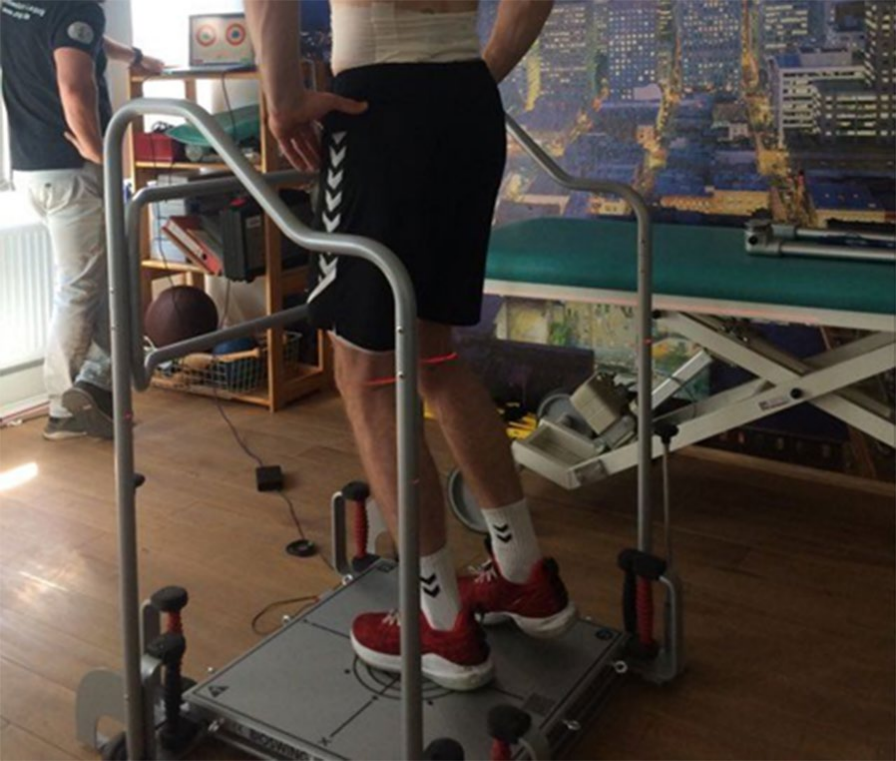
Standardized measurement in a one-legged stand on a Posturomed

Each subject completed two tests. They did the one-legged balance test on their spurring leg. Between tests, subjects rested for two minutes. At the start of the test, they had to firmly grasp their hips with their hands (Fig. 3). The one-stand leg had to be flexed at a 45° knee angle throughout the test. The angle was determined with a goniometer and marked with a skin marker in the back of the knee. The joint gap was palpated for the horizontal marker position, and the center of the horizontal marker was selected for the vertical marker. Figure 3 shows that the marker was used to check the execution standard with a cross-line laser (Bosch cross-line laser Quigo, Robert Bosch GmbH, Germany). The cross-line laser was located behind the person and was used for correction when the person left the defined position. Our balance measurement data were assessed with the Posturomed and the corresponding software (BIOSWING MircoSwing V.5.0, HAIDER BIOSWING GmbH, Germany). To determine balance ability, a device-automated score was displayed between 0 and 1000 points. 1000 points represent the highest possible score in the posturocybernetics test. The score is determined by the PC software based on the distance covered by the platform. Figure 3 illustrates standardized measurements on a Posturomed.

### Kettlebell Swing Test

**Fig. 4:**
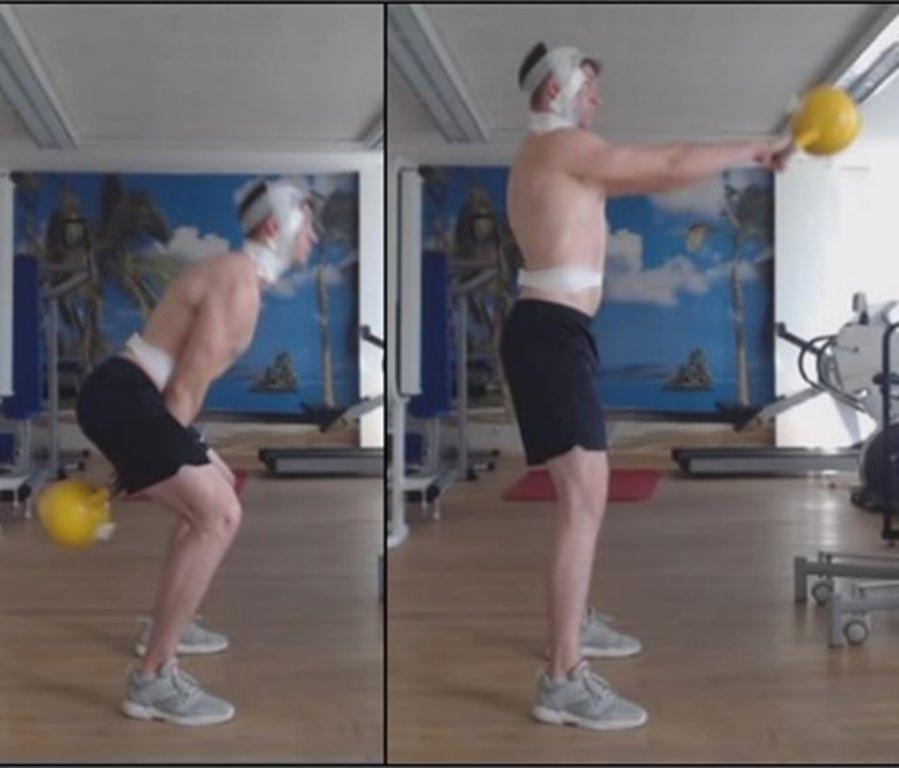
Execution of the kettlebell swing

When performing the kettlebell swing, subjects did two rounds of 15 repetitions each. They were instructed to perform the exercise at maximum speed and with a clean performance quality. They were given a three-minute rest between the two test runs. A fixed turning point in the exercise was defined by reaching shoulder height and swinging through the legs. A 16-kg cast iron kettlebell (Color Kettlebells—Vinyl, Gorilla Sports, Germany) was used. To measure the maximum and average force in watts (W), an accelerometer (Beast Sensor, Beast Technologies S.r.l., Italy) was magnetically attached to the kettlebell and fixed with tape. All data were analyzed automatically. Only repetitions 6 to 10 from each trial were included in our data analysis. Figure 4 shows the execution of the kettlebell swing.

### Counter Movement Jump

**Fig. 5:**
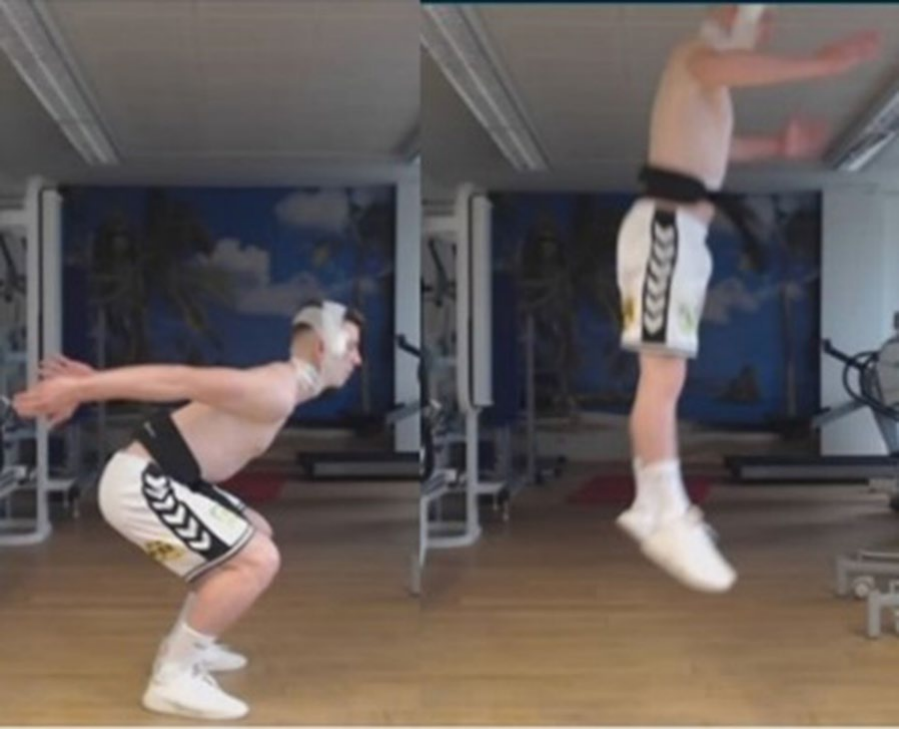
Execution of a countermovement jump

Jump height (cm) during the countermovement jump was measured via a high-speed force transducer (Achillex Jumpn'run, Xybermind GmbH, Germany). Subjects completed a total of three jumps with the intention of a maximum jump in vertical direction. In doing so, they were to actively use their arms as swinging elements. During the initial swing movement, subjects were instructed to avoid a long reversal phase to ensure fluid movement execution. The jump height was measured in centimeters. These data were evaluated using the appropriate software (Humotion Software, Xybermind GmbH, Germany). The test with the highest jump height was included in our calculation. Figure 5 shows the execution of a countermovement jump.

### EMG

The muscular activation force and activation distribution were measured electromyographically (Ultium EMG System, Noraxon, USA). Eight wireless Ultium EMG sensors (EMG sensor, Noraxon, USA) use a 24 bit and a sampling rate of up to 4000 Hz. The signal was sent directly from the point of origin to the Ultium EMG receiver via direct-function wireless technology. Noraxon Dual EMG electrodes (EMG electrodes, Noraxon, USA) with a 10 mm diameter at a 20 mm distance between the electrodes was used to record muscle activity. The experiments were filmed with a time-synchronized USB camera (CX405 Handycam EXMOR CMOS SensorThus, SONY, Japan) so that the EMG signal could be allocated precisely to the motion execution.

### Data Processing and Statistical Analysis

Raw data were processed using Noraxon MyoMuscle software (myoRESEARCH, Noraxon, USA). For the measurement with the EMG from Noraxon, rough values were processed using a high-pass filter with a high-pass frequency of 15 Hz. Subsequently, all signals were rectified and the curves digitally smoothened. The root mean square algorithm was used for 50 ms for this purpose. Amplitudes were then normalized to the mean. All data are expressed as mean value and standard deviation (SD). Data were tested for normal distribution using the Kolmogorov–Smirnov test.

The Wilcoxon rank test was used to compare group differences with the CMG and application without it. A *p* value < 0.05 was considered significant. All values are presented as means with standard deviation. GraphPad Prism 8 (GraphPad Software Inc., California, USA) was used for statistical analysis.

To evaluate muscular activation characteristics, the bilateral mean values of the respective muscles were recorded and presented as the sum mean value (*Σ*). Muscles were recorded bilaterally, and differences between right and left muscles were designated as activation symmetry (*Δ*).

## Results

12 German junior athletes (age 18.83 ± 0.39 years; height: 191.5 ± 8.05 cm; weight: 88.58 ± 9.22 kg) were included in the study. The performed exercise tests show a high reliability [24] during the repeated measurements with and without mouthguard (ICC: CMVJa r = 0.92 and r = 0.95; one-legged balance test r = 0.95 and r = 0.98 and kettlebell swing r = 0.88 and r = 0.79, respectively).

### Maximum Bite Measurement

Our maximum bite measurement results revealed no statistical significant differences in maximum activation (*Σ*) of the muscle masseter (Co 647.6 ± 212.8 µV vs. CMG 724.3 ± 257.1 µV *p* = 0.08). There were no differences in the lateral deviations (*Δ*) of the masseter muscles between the two conditions (Co 168.4 ± 154.2 µV vs. CMG 165.3 ± 128.0 µV *p* = 0.70). Maximum clenching measurements (*Σ*) of the temporalis muscle also exhibited no differences between conditions (Co 457.2 ± 135.5 µV vs. CMG 426.6 ± 169.3 µV *p* = 0.38). We observed no differences in the lateral deviations (*Δ*) in the temporalis muscle between conditions (Co 52.75 µV ± 38.39 µV vs. CMG 58.18 ± 38.39 µV *p* = 0.48).

### Results One-Legged Balance Test

We detected no significant differences in muscular activation (*Σ*) between the two conditions in any of the muscle groups tested. The balance scores did not differ either between conditions (Co 595.7 ± 171.7 vs. CMG 581.1 ± 169.9 *p* = 0.79). Table 1 shows the mean values of the muscular discrepancies.

**Table 1:** Mean and SD for the muscular balance deviations (*Δ* in µV) at Posturomed for Co and CMG

Parameters: Differences in µV	Co Mean ± SD	CMG Mean ± SD	*p* value	$\eta_{p}^{2}$
*Δ* Masseter	0.34 ± 0.5	1.42 ± 2.9	0.41	0.12
*Δ* Sternocleidomastoids	0.55 ± 0.9	1.16 ± 1.1	0.08	0.18
*Δ* Rectus femurs	0.39 ± 0.5	0.80 ± 1.1	0.30	0.10
*Δ* Erector spinae	0.82 ± 1.0	0.63 ± 1.1	0.69	0.02

* = significantly different, Co = control, CMG = custom made mouthguard, SD = standard deviation, *Δ* = muscular balance deviations, $\eta_{p}^{2}$ = part. Eta square

### Results Kettlebell Swing Test

We observed no significant differences in muscular activity (*Σ*) between the two conditions, nor did the power output in watts differ significantly between conditions (Co 671.3 ± 111.2 W vs. CMG 675.6 ± 41.7 W *p* = 0.88).

Table 2 shows the values for the differences in muscle balance (*Δ*).

**Table 2:** Mean and SD for the muscular balance deviations (*Δ* in µV) during kettlebell swing for Co and CMG

Parameters: Differences in µV	Co Mean ± SD	CMG Mean ± SD	*p* value	$\eta_{p}^{2}$
*Δ* Masseter	8.77 ± 9.3	7.33 ± 4.8	0.60	0.03
*Δ* Sternocleidomastoids	6.06 ± 5.2	4.72 ± 5.1	0.61	0.03
*Δ* Rectus femurs	4.84 ± 5.3	6.67 ± 5.4	0.21	0.14
*Δ* Erector spinae	5.33 ± 3.4	2.53 ± 1.8	0.01*	0.52

* = significantly different, Co = control, CMG = custom made mouthguard; SD = standard deviation, *Δ* = muscular balance deviations,${ }\eta_{p}^{2}$ = part. Eta square

Table 2 shows that activation of the erector spinae muscle was significantly more balanced with the use of a CMG than without a mouthguard

### Results Countermovement Jump

Table 3 shows the results of muscle activity (*Σ*) at CMJ for the performance with CMG and without, as well as the maximum jump height achieved. There are no significant differences between conditions for both muscle activation (*Σ* in µV) and jumping performance.

**Table 3:** Mean and SD of sum muscle activity (*Σ* in µV) at CMVJa for Co and CMG as well as jump height (in cm)

Parameters: Sum in µV	Co Mean ± SD	CMG Mean ± SD	*p* value	$\eta_{p}^{2}$
*Σ* Masseter	189.2 ± 59.2	158.1 ± 36.4	0.06	0.21
*Σ* Sternocleidomastoids	166.4 ± 49.1	138.3 ± 42.5	0.19	0.15
*Σ* Rectus femoris	146.7 ± 48.7	156.8 ± 84.7	0.86	0.01
*Σ* Erector spinae	193.3 ± 55.5	230.3 ± 82.8	0.12	0.13
Jump height in cm	52.5 ± 5.2	53.3 ± 4.8	0.43	0.06

* = significantly different, Co = control, CMG = custom made mouthguard; SD = standard deviation, *Σ* = sum of bilateral muscular mean values, $\eta_{p}^{2}$ = part. Eta square

Linear regression analysis shows that the masseter activity mean values of the maximum bite block measurement without CMG and the masseter activity mean values with CMG (*p* = 0.81) and without CMG (*p* = 0.36) have no detectable correlations in the CMVJa exercise.

Table 4 shows the results of the differences in symmetrical muscle activity at CMVJa for Co and CMG. Balanced EMG-induced muscle activation symmetry (*Δ* in µV) shows significant differences for the erector spinae and sternocleidomastoid muscles with CMG (Table 4).

**Table 4:** Mean and SD for the muscular balance deviations (*Δ* in µV) at the CMVJa for Co and CMG

Parameters: Differences in µV	Co Mean ± SD	CMG Mean ± SD	*p* value	$\eta_{p}^{2}$
*Δ* Masseter	31.73 ± 29.9	16.33 ± 10.5	0.12	0.20
*Δ* Sternocleidomastoids	25.48 ± 20.6	8.60 ± 9.4	0.03*	0.37
*Δ* Rectus femurs	18.75 ± 18.0	19.67 ± 13.5	0.89	0.01
*Δ* Erector spinae	37.90 ± 30.6	17.83 ± 22.3	0.03*	0.38

* = significantly different, Co = control, CMG = custom made mouthguard; SD = standard deviation, *Δ* = muscular balance deviations, $\eta_{p}^{2}$ = part. Eta square

**Fig. 6:**
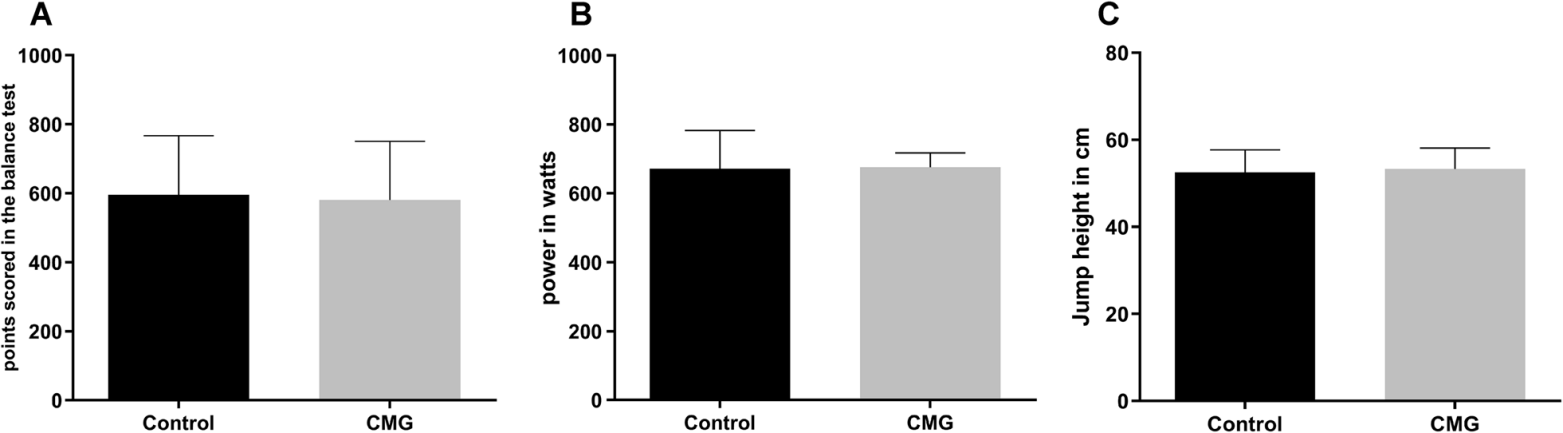
Performance tests **A** single-leg stance; **B** kettlebell swing; **C** countermovement jump

Figure 6 shows the power output for the specific tests under the CMG and no CMG conditions.

Figure 6 shows that there were no differences in the strength achievable during the single-leg stance, kettlebell swing, and CMVJa when wearing a CMG compared to the strength without a CMG.

## Discussion

Our maximum bite force measurements under resting conditions revealed no statistical differences with versus without a CMG. During the exercises, there were no differences in total muscular activation (*Σ*) between wearing a CMG and without one. However, we observed significantly less lateral deviation (*Δ*) of muscle activity during the dynamic tests with a CMG. These findings indicate more symmetric activation (*Δ*) when wearing a CMG.

### Maximum Bite Measurement

Our maximum bite force measurements showed no statistical significance, but rather a tendency toward increased activity of the muscle masseter (*Σ*) when wearing a CMG. The study by Schulze et al. [39] reported significantly more masseter activity in their maximum bite measurements with cotton rolls rather than without during forced bite clenching [38]. They hypothesized that a mouthguard is particularly beneficial for individuals with an intercuspal disorder, resulting in a loss of functional masseter muscle strength [38]. Their point of view implies the presumption of greater intercuspal distance with mouthguards and thus better activation of the jaw muscles, or the elimination of existing muscular jaw imbalances [38].

In summary, the results presented here seem to indicate that the masseter muscle’s activation (*Σ*) tends to improve with mouthguards, but we found no evidence of any improvement in the mandibular lateral deviation (*Δ*) [38].

### Exercise Measurements

The results shows that there were no differences in the strength achievable during the single-leg stance, kettlebell swing, and CMVJa [12] when wearing a CMG compared to the strength without a CMG.

Recent studies, on the other hand, have suggested that increased clenching force through remote voluntary contraction (RVC) may lead to forcing concurrent activation potentiation (CAP) to occur—thus improving an athlete’s motor performance when wearing an mouthguard [1, 2, 5, 6, 9, 38]. Busca et al. [5] demonstrated a significantly higher power and jump height in a CMVJa with a customized mouthguard. The present results show neither a significantly increased jump height nor a significantly improved muscle activation (*Σ*). The players performed the exercises without a predefined bite order; therefore, a lack of potentiation phenomenon [15, 16] could explain the unchanged performance. In this study, we did not observe higher masseter activation (*Σ*) or improved performance, so RVC is not likely to occur under these conditions. However, there was a significant improvement in muscular symmetry (*Δ*) in the CMVJa using a CMG, particularly in the neck and back muscles. The trended and noticeable improvement in the symmetry of the masseter muscles in the dynamic exercise of the CMVJa could be an indication of altered temporomandibular joint posture [38] due to the CMG and thus positive effects on the connection to the neck muscles as well as to the entire dorsal muscle chain.

The present data do not suggest any motor performance enhancement from wearing a CMG [8, 10, 22, 39]. Some studies show that forced biting, as opposed to a relaxed jaw condition, increases peripheral muscular activity with and without an mouthguard [1, 2, 15, 41]. However, Allen et al. [1] showed that peripheral muscle activity did not differ between the condition with versus without mouthguard. Another study measuring EMG showed that both muscle masseter and deltoid muscle activity were greater with mouthguards [41]. In our investigation, we studied natural jaw conditions during loading; we gave no instructions to clench the teeth. Our results show a tendency toward increased activity of the masseter muscle with a CMG when measuring the maximum bite force at rest, but no increased activation of the masseter muscle or peripheral muscles was observed during exercise performance. Based on the mean values of masseter muscle activity during measurement of maximum bite block, there was a reduction in masseter activation of approximately 78% with CMG and 71% without CMG. What we did notice was that during the CMVJa exercise, the masseter’s total activation tended to be weaker, and its lateral deviations also tended to be more reduced than when wearing the CMG. These data suggest that there is either no stronger bite force under load with mouthguards, or that athletes do not voluntarily clench their jaws during a load. Several studies have demonstrated that clenching the teeth can improve postural stability or balance control [4, 18, 19, 25, 33]. Some authors suspect the presence of ergogenic effects from wearing an mouthguard, especially during dynamic exercises demanding a great deal of force [1, 2]. We failed to detect any improved balance scores in our subjects’ single-leg stand, but we did observe significantly reduced lateral deviations (*Δ*), especially in the erector spinae muscle during the dynamic exercises. We also noted better balanced control of the sternocleidomastoids muscle during a CMVJ together with wearing a CMG. There is some evidence that dental protection induces jaw modifications that favor peripheral muscle activity [2, 6, 13, 38]. We speculate that the masseter muscle’s reduced lateral deviation (*Δ*) during the CMVJ, like the back muscles’ reduced lateral deviation (*Δ*), is most likely indicative of muscular activation’s forced symmetry both centrally and peripherally while wearing a CMG. More balanced control of the antagonistic and agonistic musculature can but need not necessarily be considered a performance-enhancing component.

The authors of this paper presume that there is a possible relationship between the tendency for increased activation of the masseter muscles under forced clenching and significantly more symmetric activation (*Δ*) in the lumbar spine during dynamic exercises using a CMG. As stronger activity of the masticatory muscles under load has not been detected [38, 41], this might imply that subjects do not clench their jaws more during either dynamic or static exercises.

In short, there is no evidence of improved motor performance [8, 22, 28, 39] nor any increased activation of peripheral muscles [1]—findings that argue against concurrent activation potentiation and more in favor of improved symmetric stimulation (*Δ*), especially of the back muscles under these conditions.

### Study Limitations

Since the participants were professional handball players, only max one free training day could be used for the examination. The sample size is small, and only male participants were enrolled; therefore, the interpretability and generalizability of the results are limited. However, this trial is the largest randomized crossover study performed to date regarding the acute neuromuscular response due to wearing a mouthguard. In the present study, we did not use a MVC test. When measuring MVC, the subject would have to be tested in static position. In particular, it is practically impossible to simulate maximum voluntary contraction in the sternocleidomastoids and erector spinae. Furthermore, we hypothesized that during dynamic exercises, athletes are creating greater muscular activity and that, by normalizing to the MVC, a cut-off effect could occur [30]. Surface EMG represents specific challenges in dynamic exercises, such as the degree of nonstationarity of the signal or the relative displacement of the electrodes with respect to the origin of the action potentials. We thought to minimize these limitations by highly standardized electrode positions and adding a video-based time-related analysis to assess the onset of muscle activation [17].

## Conclusion

There was neither an evident effect to improve motor performance nor increased muscle activity (*Σ*) by wearing a CMG, but significantly more balanced activity (*Δ*) of cervical and dorsal muscles was observed under dynamic conditions. Our study results suggest that a CMG has no negative effects on the motor performance and partial positive effects on the balance of muscle activation. We believe that these effects deserve further investigation.

## Data Availability

The datasets used and/or analyzed during the current study are available from the corresponding author on reasonable request.

## Abbreviations

CAP: Concurrent activation potentiation
CMG: Customized mouthguard
CMVJa: Countermovement jump with arms
Co: Control
EMG: Electromyography (Ultium EMG System, Noraxon, USA)
KBS: Kettlebell swing
RVC: Remote voluntary contraction
sEMG: SinfoMed electromyography
SLS: Single-leg stand
W: Watt
